# Severe loss of appetite and refusal to eat as severe side effect of glycerol phenylbutyrate

**DOI:** 10.1002/jmd2.12286

**Published:** 2022-09-30

**Authors:** Sarah Catharina Grünert, Anke Schumann, Ute Spiekerkoetter

**Affiliations:** ^1^ Department of General Pediatrics, Adolescent Medicine and Neonatology Medical Center‐University of Freiburg, Faculty of Medicine Freiburg Germany

**Keywords:** glycerol phenylbutyrate, loss of appetite, ornithine transcarbamylase deficiency, Ravicti®, urea cycle defect

## Abstract

Glycerol phenylbutyrate (GPB) is an ammonia scavenger drug commonly used in the therapy of patients with urea cycle defects. Reported side effects include body odor, abdominal pain, nausea, burning sensation in mouth, vomiting, and heartburn. We report on a 3‐year‐old late diagnosed female patient with ornithine transcarbamylase deficiency that experienced severe loss of appetite under treatment with GBP. Due to catabolism (calory intake about 400 kcal/day) and the associated risk of metabolic decompensation, GBP treatment was discontinued. Her appetite and eating behavior normalized within 1 day after discontinuation of GBP and switch to sodium benzoate. Our case demonstrates that GBP can cause severe loss of appetite that may put patients at risk of metabolic decompensation and require discontinuation of therapy.

**Synopsis**

Complete loss of appetite can be a severe side effect of glycerol phenylbutyrate that may require discontinuation of therapy.

**Abbreviations**
GPB: glycerol phenylbutyrate
NaPBA: sodium phenylbutyrate
OTC: ornithine transcarbamylase
UCD: urea cycle defect

## 1 INTRODUCTION

Glycerol phenylbutyrate (GPB) has become a first line treatment for patients with urea cycle defects (UCDs) either as monotherapy or in combination with other ammonia scavengers.^1^ The most commonly reported side effects of GPB comprise body odor, abdominal pain, nausea, burning sensation in mouth, vomiting, and heartburn.^2^ In comparison to sodium phenylbutyrate (NaPBA), GPB is not only more palatable but also seems to have less side effects.^2^ We report on a 3‐year‐old girl with complete loss of appetite under GPB treatment that necessitated the discontinuation of treatment.

## 2 CASE PRESENTATION

The patient is the first child of non‐consanguineous parents. She was diagnosed with ornithine transcarbamylase (OTC) deficiency at the age of 2 when she presented with recurrent vomiting and subacute liver failure (GPT 1500 U/L, normal <35 U/L; GOT 940 U/L, normal <35 U/L; INR 2.3, normal 0.85–1.15; ammonia 150 μmol/L, normal 15–70 μmol/L). The diagnosis was made biochemically and confirmed by mutation analysis of the *OTC* gene. Both the patient and her mother were found to be heterozygous for the c. 626C>T; p.Ala209Val variant in the *OTC gene*. Our patient recovered quickly from the acute episode. A low protein diet with a total protein intake of 1.3 g/kg body weight per day was recommended together with the administration of GPB (Ravicti®, 3 × 1.5 ml, 9.0 ml/m^2^). Essential amino acids were not supplemented. Under this treatment she was metabolically stable and her ammonia and glutamine concentrations in serum always remained within the normal range. Shortly after the initiation of GPB, however, the mother reported that the girl who had always been a good eater with respect to both her appetite as well as the diversity of her diet started to become selective with food. She did not complain about abdominal pain, but had a complete loss of appetite resulting in refusal to eat and necessity of supplementation with maltodextrin to provide extra energy. She also refused many foods that she had liked before. Although the parents tried to motivate her, her caloric intake was only about 400 kcal/day resulting in a weight loss of about 1.5 kg. Despite this, ammonia levels were always in the normal range. As loss of appetite is described as a possible side effect of GPB, the medication was discontinued and replaced by sodium benzoate. This led to an immediate normalization of her eating behavior within 1 day and a subsequent weight gain of about 1 kg within 4 weeks.

## 3 DISCUSSION

GPB has recently become a widely available pharmacological treatment for patients with UCDs. Beside its efficacy in lowering ammonia levels^3^ and promoting metabolic stability,^4^ its better taste and easier administration method compared with NaPBA are important advantages especially for the treatment of pediatric patients. Yeowell et al. have recently performed a study to assess the burden of pharmacological treatment and the effects of the transition to GPB on health‐related quality of life.^5^ They interviewed nine caregivers of children living with a UCD regarding their experiences of pharmacological treatment and reported that medication administration was perceived to have improved with GPB, alleviating distress for both carer and child.

To assess self‐reported treatment‐associated symptoms among patients with UCDs, Nagamani et al. performed a prospective data collection among patients who participated in clinical trials of GPB^2^ and who were previously treated with NaPBA (*n* = 100). Compared to NaPBA, GPB lead to a significant and persistent reduction of treatment‐associated symptoms. While decreased appetite was reported by 10 patients (10%) under treatment with NaPBA, only 1 patient (1.1%) was still complaining about this symptom 9–12 months after the switch to GPB. Although severe loss of appetite seems to be a rare treatment‐associated symptom of GPB, our case shows that this severe side effect may require discontinuation of therapy, as catabolic episodes put patients with urea cycle defects at risk of metabolic decompensation. Although we have not performed a gastroscopy in our patient, an acute gastritis as cause of the gastrointestinal symptoms is rather unlikely due to the fact that the symptoms were reversible within a very short time after discontinuation of GPB treatment.

## CONFLICT OF INTEREST

The authors declare that they have no competing interests.

## AUTHOR CONTRIBUTIONS

All authors were involved in the clinical management of the patient. Sarah Catharina Grünert drafted the manuscript. Anke Schumann and Ute Spiekerkoetter critically revised the manuscript. All authors approved the final version of the manuscript.

## CONSENT FOR PUBLICATION

The patients' parents gave their informed consent for the publication of this case report.

## ETHICS STATEMENT

Not applicable.

## Data Availability

Not applicable.
